# Integrating Sensor Ontologies with Niching Multi-Objective Particle Swarm Optimization Algorithm

**DOI:** 10.3390/s23115069

**Published:** 2023-05-25

**Authors:** Yucheng Zhuang, Yikun Huang, Wenyu Liu

**Affiliations:** 1 Fujian Provincial Key Laboratory of Big Data Mining and Applications, Fujian University of Technology, No. 69 Xuefu South Road, Minhou, Fuzhou 350118, China; zhuangyucheng2021@163.com; 2 Concord University College, Fujian Normal University, No. 68 Xuefu South Road, Minhou, Fuzhou 350117, China; 3 School of Computer Science and Mathematics, Fujian University of Technology, No. 69 Xuefu South Road, Minhou, Fuzhou 350118, China; wenyuliu1983@hotmail.com

**Keywords:** sensor ontology matching, multi-modal optimization, niching multi-objective particle swarm optimization algorithm, Ontology Alignment Evaluation Initiative

## Abstract

Sensor ontology provides a standardized semantic representation for information sharing between sensor devices. However, due to the varied descriptions of sensor devices at the semantic level by designers in different fields, data exchange between sensor devices is hindered. Sensor ontology matching achieves data integration and sharing between sensors by establishing semantic relationships between sensor devices. Therefore, a niching multi-objective particle swarm optimization algorithm (NMOPSO) is proposed to effectively solve the sensor ontology matching problem. As the sensor ontology meta-matching problem is essentially a multi-modal optimization problem (MMOP), a niching strategy is introduced into MOPSO to enable the algorithm to find more global optimal solutions that meet the needs of different decision makers. In addition, a diversity-enhancing strategy and an opposition-based learning (OBL) strategy are introduced into the evolution process of NMOPSO to improve the quality of sensor ontology matching and ensure the solutions converge to the real Pareto fronts (PFs). The experimental results demonstrate the effectiveness of NMOPSO in comparison to MOPSO-based matching techniques and participants of the Ontology Alignment Evaluation Initiative (OAEI).

## 1. Introduction

Sensors have been widely applied in various fields such as environmental protection and remote sensing technology to meet the needs of information sharing and transmission [1,2]. Sensor web (SW) is a distributed network composed of many sensor nodes that collect massive amounts of data from various applications [3]. However, inconsistent semantic information among sensor data in different applications may hinder communication among sensors. To achieve semantic information sharing, it is necessary to use Semantic Sensor Web (SSW). Sensor ontology is the core of SW and enables sensor semantic information sharing and data interoperability [4]. However, heterogeneous problems can arise from different ways of describing sensor data in different applications [5]. Sensor matching technology establishes semantic relationships between heterogeneous ontologies to achieve information sharing in SW.

Similarity measures are the core of sensor ontology matching technology used to evaluate the degree of similarity between entities of sensor ontologies. Ontology meta-matching technology is a hot research direction for ontology matching, with a focus on choosing appropriate similarity measures, assigning appropriate weights and threshold values to the generated similarity matrix, and achieving the final matching result [6]. In addition, the selection process of weight needs to trade off the two measures of matching results, namely, recall and precision. In the process of ontology matching, how to filter the unreal result through the appropriate threshold is the main key. Since the selection of weight and threshold affects the ontology matching results, it is usually modeled as an optimization problem.

PSO is a classical meta-heuristic swarm intelligence algorithm proposed by Eberhart and Kennedy [7]. PSO is suitable for solving various optimization problems due to its fast convergence speed and strong robustness. According to the characteristics of the ontology meta-matching problem, PSO can successfully solve it. First, PSO is able to adjust the optimization objective to satisfy the needs of decision makers. Second, PSO can handle large-scale problems, such as large-scale ontology matching. In addition, PSO has fast convergence speed that reduces the time cost and improves the efficiency of ontology matching. Although PSO can effectively solve the ontology matching problem, it only optimizes one of the matching evaluation metrics, namely, recall or precision. The matching quality metrics—recall and precision—are two conflicting objectives to evaluate the matching results. To achieve better matching quality, both metrics should be optimized simultaneously. Therefore, this paper utilizes multi-objective particle swarm optimization (MOPSO) to improve the quality of ontology alignment, inspired by its success in solving the ontology meta-matching problem. In recent years, because of its effectiveness in practical applications, the multi-objective particle swarm optimization algorithm has attracted the attention of scholars. Liu et al. [8] proposed a multi-objective particle swarm optimization algorithm based on network embedding for complex network community detection, combining a network embedding method with a multi-objective particle swarm optimization algorithm to find a better optimal solution. In addition, multi-objective particle swarm optimization with fuzzy cost has demonstrated its effectiveness in feature selection [9]. By incorporating a fuzzy cost function and multi-objective particle swarm optimization, this algorithm can effectively address the challenges of noise and uncertainty in feature selection while improving population diversity. However, traditional MOPSO has three basic shortcomings when it comes to solving ontology meta-matching problems, leading to an inability to effectively solve them. Firstly, the same value exists in the objective space, corresponding to multiple solutions in the decision space in the ontology meta-matching problem. This kind of problem is essentially a multi-modal optimization problem (MOP). The traditional MOPSO carries out information sharing among particles to find the global optimal solutions, limiting the search direction of the algorithm, and often can only obtain one global optimal value, which is not suitable for solving MOP. MOPSO can find a certain solution, but in reality, the remaining optimal solution may be more valuable to the decision makers. Secondly, classical MOPSO has two basic problems when solving the ontology matching problem: how to make the solutions converge to the Pareto front (PF) quickly and how to make the solutions uniformly distributed on the PF. The local best particles and the global best particles used to guide the update of the solution greatly compress the search space of the solutions. MOPSO has fast convergence speed and uneven distribution, which will reduce the quality of the solution. To obtain better quality ontology matching, it is necessary to overcome the shortcomings of MOPSO. Specifically, how to select the leader solution and improve the population diversity are very important for the performance of MOPSO. Finally, when facing multi-modal ontology meta-matching problems with multiple global optimal solutions, MOPSO tends to fall into local optima due to the limitation of particle learning when updating the position, which reduces the alignment’s quality.

To overcome the three defects mentioned above, this paper proposes a niching multi-objective particle swarm optimization algorithm (NMOPSO) for effectively solving the sensor ontology meta-matching problem. The contributions of this paper are as follows:

- First, the niching strategy is introduced into MOPSO in this work to ensure the particles can maintain their own niche while simultaneously eliminating individuals with lower fitness values to enhance the diversity of the decision space since the sensor ontology meta-matching problem is a multi-modal multi-objective problem with multiple global optimal solutions.
- Second, the diversity-enhancing strategy is employed to balance the convergence and distribution of the algorithm when selecting the elite solutions, and random particles are selected from the elite set as candidate solutions to solve the problem for which MOPSO cannot guarantee the population diversity. In addition, by comparing the two metrics of sensor ontology meta-matching results, i.e., recall and precision, this strategy can make the solution with poor results learn from the solution with better ones and enhance the diversity of the population to improve the matching quality.
- Finally, the opposition-based learning strategy is applied to MOPSO’s update component to find the solutions with better fitness values, which helps to further make the solution approach the frontier and enhance the alignment’s quality.

The paper is structured as follows: Section 2 studies the development of existing ontology matching techniques and the definition of the sensor ontology matching problem. Section 3 presents the implementation of NMOPSO. The experimental results and discussions are presented in Section 4, and Section 5 concludes the work and identifies directions for subsequent work.

## 2. Materials and Methods

### 2.1. Literature Review of Ontology Matching Technique

Generally, the techniques to solve the ontology matching problem fall into two main categories, i.e., machine learning (ML)- and swarm intelligence (SI)-based ontology matching techniques. The ML-based matching technique models the ontology matching problem as a classification or regression problem. Common methods include support vector machine (SVM) [10], decision tree (DT) [11], logistic regression (LR) [12], etc. The SVM-based matching method [10] solves the dependence of the learning-based matching method on the instances in the ontology by non-instance learning. The DT-based matching method [11] uses the advantages of classifiers to match instances of source and target ontologies without the aid of external dictionaries. The LR-based matching method [12] selects five different similarity measures and predicts the similarity of the matching by the trained regression model. These ML-based matching techniques require training models, which often take a long time to solve the ontology matching problem. Solving ontology matching is a time-consuming and complex task. To overcome the time-consuming problem of training models, SI-based ontology matching techniques have been paid attention by many scholars.

In recent years, the swarm intelligence algorithm has become a suitable method to solve the ontology matching problem. Genetics for Ontology Alignments (GOAL) [6] is the first matching system to solve the ontology matching problem using the evolutionary algorithm (EA). Alexandru-lucian and Iftene [13] further used EA to optimize the parameters and thresholds and filtered unconvincing results to improve alignment’s quality. Later, Acampora et al. [14] continued to improve EA through the local search strategy to improve the quality of matching. Xue and Wang [15] used a new metric as a fitness function and simultaneously determined the weights required by several entities’ pairs in the matching process to approximately measure the alignment’s result. The computational cost of these methods is higher for storing the similarity matrices, and makes them unable to obtain high-quality alignment. To solve this problem, Alves et al. [16] proposed a hybrid genetic algorithm that combines EA with local search strategies to match entities and determine optimal concept mapping. More recently, Chu et al. [17] proposed a compact evolutionary algorithm (CEA) and established a new model for the ontology matching problem in vector space.

The particle swarm optimization algorithm (PSO), as a classical swarm intelligence algorithm, is widely used in the field of ontology matching because of its advantages of fast convergence and simple implementation. Bock and Hettenhausen [18] proposed a discrete PSO to solve the ontology matching problem, which is a population-based method and does not need to calculate the large similarity matrices. Yang et al. [19] used PSO to optimize parameter configurations in the matching process. Marjit et al. [20] introduced a local search process into PSO to improve the efficiency of solving the ontology meta-matching problem. Huang et al. [21] introduced a compact particle swarm optimization (cPSO) to enhance the quality of matching results when solving the sensor ontology matching problem. Zhu et al. [22] proposed a simulated annealing PSO for ontology matching, which enhanced the local search capability of the algorithm.

However, these methods are based on single-objective PSO to solve the ontology matching problem, tending to fall into local optima easily and be unable to obtain better alignment. In order to address the impact of optimizing only a single objective on the matching results, Semenova and Kureychik [23] applied MOPSO to solve the ontology matching problem. Recently, Wang et al. [24] proposed a compact MOPSO to solve the hydrological ontology matching problem based on the swarm intelligence approach of compact technology. Meanwhile, Xue et al. [25] determined the knee solution by using the max–min strategy and proposed a compact MOPSO to solve the biomedical ontology matching problem. Geng and Lv [26] used the sparsity and density indices in MOPSO to address the sensor ontology meta-matching problem, succeeding in enhancing the alignment’s quality.

Existing experiments show that ontology meta-matching is a multi-modal problem with multiple global optimal solutions. There is currently no ontology meta-matching system based on the PSO model for the meta-matching problem, which is a multi-modal problem and can reduce the efficiency of the algorithm. The overall goal of this paper is to enhance the efficiency of the algorithm by developing an improved MOPSO to optimize the ontology alignment’s quality and construct a multi-objective model to solve the multi-modal multi-objective problem.

### 2.2. Sensor Ontology Furthermore, Ontology Alignment

**Figure 1:**
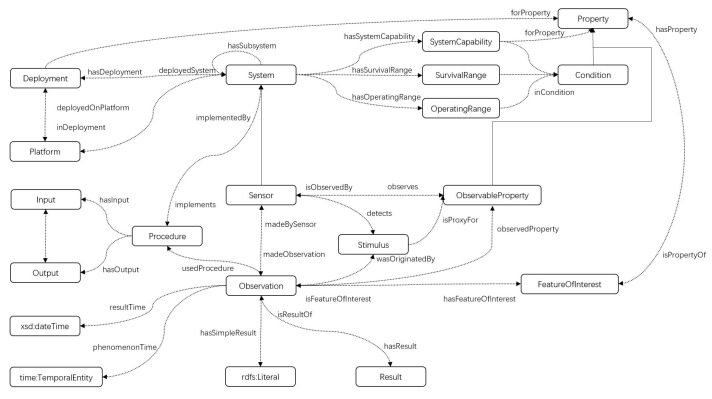
An example of sensor ontology.

The sensor ontology is defined as a 3-tuple O=(C,P,I), where *C*, *P*, and *I* denote objects, relations, and instances, respectively. Figure 1 illustrates a sensor ontology. Rounded rectangles represent classes, and bidirectional arrows represent relationships that two objects have with each other, for example, “Sensor” and “ObservableProperty”, where Sensor observes ObservableProperty, and in turn, ObservableProperty is Observed by Sensor. A one-way arrow represents a property between two objects, for instance, the relation between “Procedure” and “Input” means Procedure has Input.

**Figure 2:**
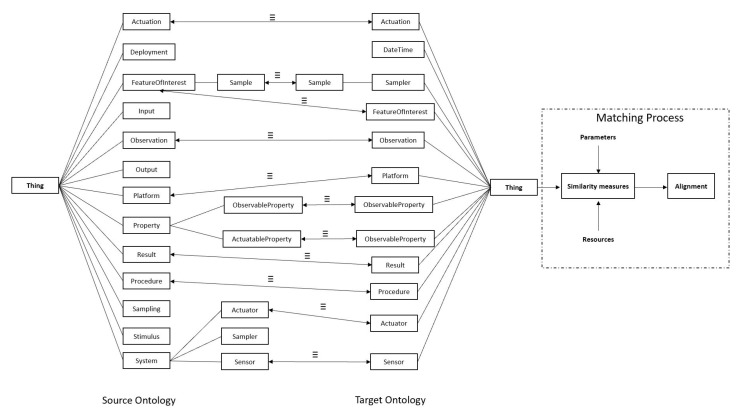
The process of sensor ontology alignment.

Since different designers may have different descriptions of sensor ontology, ontology heterogeneity will be generated. To address the heterogeneity problem, it is necessary to carry out the ontology matching process and find out the relations between ontology concepts. Figure 2 presents the process of sensor ontology alignment. Rectangles represent entities, and the symbol “≡” connecting entities with bidirectional arrows indicates that the entities are equivalent relations. Equivalent matching entity pairs in the figure include Actuation ≡ Actuation, Sample ≡ Sample, FeatureOfInterest ≡ FeatureOfInterest, Observation ≡ Observation, Platform ≡ Platform, ObservableProperty ≡ ObservableProperty, ActuatableProperty ≡ ObservableProperty, Procedure ≡ Procedure, Actuator ≡ Actuator, and Sensor ≡ Sensor. The set of these equivalence relations is the matching result. Sensor ontology includes source ontology and target ontology. The matching process requires parameters and external resources, and the corresponding similarity matrices are generated by the appropriate similarity measures to obtain the final ontology alignment.

The matching result’s metrics precision and recall are conflicting objectives in the ontology meta-matching problem. When one objective increases, the other objective decreases. The definitions are as follows:

(1) $$recall=\frac{\left|R⋂A\right|}{\left|R\right|}$$

(2) $$precision=\frac{\left|R⋂A\right|}{\left|A\right|}$$

where *R* and *A* denote the reference alignment and the ultimate alignment, respectively, which are formulated by domain experts. If only a single objective is optimized in the matching process, that is, only recall or precision is optimized, the perfect matching results cannot be obtained because the two objectives conflict with each other. Therefore, it is necessary to optimize recall and precision at the same time to improve the matching quality. On the basis of this, the mathematical definition of the multi-objective optimization model for sensor ontology meta-matching problem is as follows:

(3) $$\left\{\begin{array}{c} \max f(Weight,Threshold)=(\mathrm{recall}(Weight,Threshold),\mathrm{precision}(Weight,Threshold))\\ \;s.t.\;Weight={\left(w_{1},w_{2},\ldots \right)}^{T},Threshold\in \left[0,1\right]\\ \sum_{i=1}^{N}w_{i}=1,\;w_{i}\in \left[0,1\right] \end{array}\right.$$

where Weight and Threshold represent the aggregating weights set and the threshold, respectively. $w_{i}$ is the weight value of the *i*th similarity measure and *N* represents the number of similarity measures.

## 3. Proposed Method

**Figure 3:**
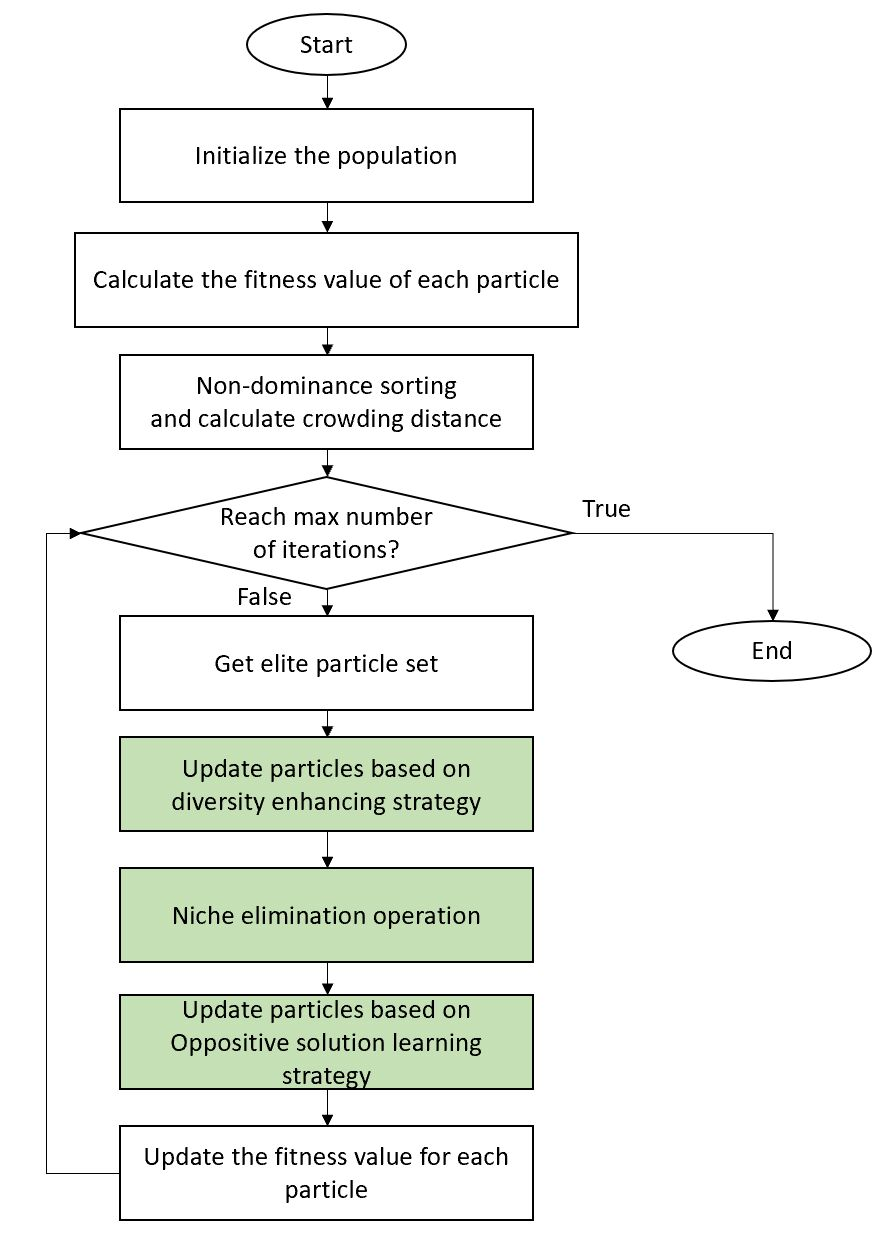
The flowchart of NMOPSO.

To solve the problem of multi-modal multi-objective sensor ontology meta-matching with multiple global optimal solutions, it is necessary to keep the diversity of the population in the objective space and decision space. For this purpose, this paper proposes an improved MOPSO based on niche and an opposition-based learning strategy, called NMOPSO. In the proposed method, two particles were randomly selected from the elite set for pairing competition through the diversity-enhancing strategy to ensure the diversity in the objective space. The particle with the better fitness value is selected as the winner particle to guide the particle with the worse fitness value to update and obtain better ontology meta-matching results. Since the sensor ontology meta-matching problem is a multi-modal problem, the niche elimination strategy is used in the updating process. By comparing the fitness between particles and setting the penalty coefficient for particles with poor fitness value, the particles with better fitness can be preserved in the evolutionary process to better maintain the diversity of the population in the decision space. Moreover, to further approximate the optimal solutions, the opposition-based learning strategy is introduced to update the particles in the evolution process to improve the quality of sensor ontology meta-matching. The flowchart for NMOPSO is shown in Figure 3, and the components will be explained in detail in the following subsections. The main parts of the algorithm are marked and distinguished, and will be described in the next section.

### 3.1. Encoding Mechanism

In this paper, the decimal coding mechanism is used to encode particles. Because decimal coding is easy to understand and can correspond to the threshold and weight information of particles, it is more suitable for the sensor ontology meta-matching problem. Algorithm 1 gives the pseudo-code of the encoding mechanism. Each particle consists of aggregating weights and a threshold. First, *n* real numbers randomly in [0,1] are generated, denoted as $r_{1}$, $r_{2},\ldots ,r_{n-1}$, $r_{n}$; then, the first n−1 cut points $r^{\prime }$ ={$r_{1}^{\prime }$, $r_{2}^{\prime },\ldots ,r_{n-1}^{\prime }$} are sorted in ascending order. Finally, the aggregating weights are calculated as follows:

(4) $$w_{i}=\left\{\begin{array}{cc} r_{1}^{\prime }, & t=1\\ r_{i}^{\prime }-r_{i-1}{}^{\prime }, & 1<i<n\\ 1-r_{n-1}^{\prime }, & i=n \end{array}\right.$$

**Algorithm 1** Encoding Mechanism**Input:** The particle *p* = {$r_{1}$, $r_{2},\ldots ,r_{n-1}$,threshold};**Output:** The encoded particle $p^{\prime }$ = {$w_{1}^{\prime }$, $w_{2}^{\prime },\ldots ,w_{n}^{\prime }$,threshold};   1:** Encoding **   2:Generate n real numbers randomly in [0, 1], denoted as $r_{1}$, $r_{2},\ldots ,r_{n-1}$, $r_{n}$;   3:Sort the first n−1 cut points $r^{\prime }$ ={$r_{1}^{\prime }$, $r_{2}^{\prime },\ldots ,r_{n-1}^{\prime }$} in ascending order   4:**for ***i* = 1; *i* < *n*; *i* ++ **do**   5:   **if** *i*=1 **then**   6:     $w_{i}$ = $r_{1}^{\prime }$   7:   **else** **if** *i* = *n* **then**   8:     $w_{i}$ = 1$-r_{n-1}^{\prime }$;   9:   **else** 10:     $w_{i}$ = $r_{i}^{\prime }-r_{i-1}^{\prime }$; 11:   **end if** 12:**end for****return** The encoded particle $p^{\prime }$;

### 3.2. Diversity Enhancing Strategy

MOPSO uses an external archive to store individual and global optimal solutions during evolution. Each particle updates its own velocity and position according to the individual and the global optimal solutions. In addition, classic MOPSO has mediocre performance when dealing with optimization problems with a large number of local optimal solutions, such as the sensor ontology meta-matching problem [27]. In order to solve the influence of individual and global optimal solutions on MOPSO, this work uses diversity-enhancing strategy to guide particle updating to trade off the MOPSO’s convergence and diversity, and utilizes it to solve sensor ontology meta-matching problem.

The diversity-enhancing strategy randomly selects particles from the elite set for pairwise competition during the evolution process. The particles with better recall and precision values are selected as the winner particles, and the particles with worse ones are guided to update. The update formula for particles with lower fitness values is as follows:

(5) $$\begin{array}{c} V_{l,k}\left(t+1\right)=c_{1}V_{l,k}\left(t\right)+c_{2}\left(X_{w,k}\left(t\right)-X_{l,k}\left(t\right)\right) \end{array}$$

(6) $$\begin{array}{c} X_{l,k}\left(t+1\right)=X_{l,k}\left(t\right)+V_{l,k}\left(t+1\right) \end{array}$$

where $c_{1}$ and $c_{2}$ are the weights; *k* stands for the *K*th round of competition; *i* refers to the *i*th particle; *t* denotes the generation; and $X_{w,k}\left(t\right)$ and $X_{l,k}\left(t\right)$ are the position variables of particles with higher and lower fitness values, respectively. It is evident from the update formula of the particles with lower fitness in the K-round competition that the particle position update formula using the diversity-enhancing strategy is the same as that of classic MOPSO. The first part of the speed update formula is consistent with the MOPSO. The second part gets rid of the influence of individual and global particles and uses particles with higher fitness to guide the evolution of the population.

### 3.3. Niching Strategy

The sensor ontology meta-matching problem is a multi-modal and multi-objective problem with multiple objectives and multiple optimal solutions. The key to solving this kind of problem is to balance different optimal solutions and provide decision makers with a set of optimal solutions that trade off multiple objectives. At present, in the field of swarm intelligence, the niching strategy is an effective method to solve multi-modal optimization problems.

Niching strategy was proposed by Cavicchio in 1970, and its application to the swarm intelligence algorithm can effectively solve multi-modal optimization problems [28]. The main idea of the niching strategy is to divide the population into several sub-populations, and each sub-population maintains its own niche, which can be applied to the PSO algorithm to maintain the diversity of the population.

In this work, the niching strategy is applied into MOPSO to solve the sensor ontology meta-matching problem. In details, the Euclidean distance between the particles in the population is calculated and the evaluation metrics of the ontology matching result, i.e., the recall and precision of the two particles, are compared, and the penalty function is assigned to individuals with poor fitness values. The mathematical definition of the distance between particles is as follows:

(7) $$\begin{array}{c} d_{ij}^{d}=∥p_{i}^{d}-p_{j}^{d}∥=\sqrt{\sum_{k=1}^{N}{\left(p_{i}^{d}-p_{j}^{d}\right)}^{2}} \end{array}$$

where $d_{ij}^{d}$ is the distance between particle *i* and particle *j*; *d* denotes the dimension of the problem; $p_{i}$ and $p_{j}$ are *i*th particle and *j*th particle, respectively; *N* is the number of particles in the niche. The mathematical definition of the penalty function is as follows:

(8) $$\begin{array}{c} p_{i}\left(fit\right)=p_{i}\left(fit\right)\times punishQuo \end{array}$$

where $p_{i}\left(fit\right)$ is the *i*th particle with a lower fitness value; punishQuo is a random number between 0 and 1. In the process of evolution, the possibility of the particles that impose the penalty function being eliminated increases, which maintains the diversity of the population. For the multi-modal sensor ontology meta-matching problem, each peak has a certain number of particles to ensure the global exploration performance of the algorithm.

### 3.4. Opposition-Based Learning Strategy

Opposition-based learning (OBL) was first proposed by Tizhoosh in 2005 [29]. The main idea is to generate the solutions with opposite position for the current solution position, which is conducive to improving the self-learning ability of the individual in the swarm intelligence algorithm and finding a solution with better fitness. It has been shown that the opposite solution is better for approximating the optimal solution than the randomly initialized solution [30]. OBL is often applied to the multi-objective optimization problem to further improve the performance of proposed algorithms. Therefore, in this paper, the opposition-based learning strategy is introduced into the particle update process to further improve the quality of the solutions.

According to the characteristics of the sensor ontology meta-matching problem, the OBL strategy is introduced into the update process of the MOPSO in this work. The upper and lower bounds are defined by the dimension of the sensor ontology meta-matching problem, and the opposite solutions generated in the updating process provide more solutions in the objective space, which provides more solutions for solving the multi-modal ontology meta-matching problem scheme. The opposite solution is specifically defined as follows:

(9) $$\begin{array}{c} X_{i}{\left(opp\right)}^{d}={upp}^{d}+low^{d}-X_{i}^{d} \end{array}$$

where *d* is the dimension of the problem; *i* is the *i*th particle in the population; and ${upp}^{d}$ and $low^{d}$ are the upper and lower bounds of the solution, respectively. $X_{i}^{d}$ is the current position of the *i*th particle; $X_{i}{\left(opp\right)}^{d}$ is the position of the *i*th-generated opposite solution.

### 3.5. The Pseudo-Code of Niching Multi-Objective Particle Swarm Optimization Algorithm and Complexity Analysis

The pseudo-code of NMOPSO is presented in Algorithm 1. NMOPSO first initializes the particles and calculates the fitness value of each particle. The elite particle set *L* can be obtained by calculating the crowding distance and non-dominance sorting of the solutions. In the evolutionary process, two particles are randomly selected from the elite set for pairwise competition, and the two objective function values of particles *a* and *b* are compared. If the two values of particle *a* are greater than that of particle *b*, *a* is the winner particle and guides the update of *b*. According to the formula, the Euclidean distance between two particles is calculated, the particle with lower fitness value is penalized, and the particle is updated. Then, the oppositive solution is generated for the particle based on the opposition-based learning strategy, and the particle is updated.
**Algorithm 2** The pseudo-code of niching multi-objective particle swarm optimization algorithm **Input:** source and target ontologies, $O_{1}$ and $O_{2}$; number of iterations, *N*; population size, *n*; dimension of problem, dim; upper and lower bound, upp and low;particle’s current position, *X*; particle’s current velocity, *V*; elite particle set, *L*;**Output:** winner particle $P_{win}$;   1:** Initialization **   2:initialize generation *t* = 0;   3:calculate particle’s fitness value $P_{recall}$ and $P_{precision}$   4:**for***i* = 0; *i* < dim; *i* ++ **do**   5:   V[i] = random(0, 1);   6:   X[i] = random(0, 1);   7:**end for**   8:NonDominatedSort();   9:calculateCrowdingDistance(); 10:sortFronts(); 11:get elite particle set *L*; 12:** Evolution **; 13:**while** *t* < *N* **do** 14:   select two particles *a* and *b* randomly from the elite set *L*; 15:   $P_{recall}$ and $P_{precision}$ of particle *a* and *b* are calculated, respectively; 16:   $\left[P_{win},P_{loser}\right]$ = compete(*a*, *b*); 17:   **if** $P_{\mathrm{recall}\;}\left(a\right)>P_{\mathrm{recall}\;}\left(b\right)\;\mathrm{and}\;P_{\mathrm{precision}\;}\left(a\right)>P_{\mathrm{precision}\;}\left(b\right)$ **then** 18:     $P_{win}$ = *a*; 19:   **else** 20:     $P_{win}$ = *b* 21:   **end if** 22:   ** Update **; 23:   $V_{P_{\mathrm{loser}\;}}{}^{\prime }$← update $P_{loser}$’s velocity according to the formula (5); 24:   $X_{P_{\mathrm{loser}\;}}{}^{\prime }$← update $P_{loser}$’s position according to the formula (6); 25:   ** Niche elimination operation **; 26:   calculate the Euclidean distance between two particles according to the formula (7); 27:   update particle’s fitness value by penalizing particles with lower fitness values based on the niche elimination strategy according to the formula (8); 28:   ** Opposition-based learning strategy **; 29:   update particles based on opposition-based learning strategy according to the formula (9); 30:   update particle’s fitness value $P_{recall}$ and $P_{precision}$; 31:   *t* = *t* +1; 32:**end while****return **$P_{win}$;

The proposed method uses a diversity-enhancing strategy to guide the evolution of the population, in which the elite particles of each generation are selected by the pairs of the particles of the current generation to guide the update of the population, so no additional external archive is required to keep the non-dominance solutions. Provided that the population size is *n* and the number of objective functions is *m*, NMOPSO’s time complexity consists of two components: The first part is the population initialization, and the time complexity is O(n). The second part is the updating strategy of particles. This consists of non-dominance sorting, a diversity-enhancing strategy, a niche elimination strategy, and an opposition-based learning strategy. The time complexity of non-dominance sorting is $O(m\times n^{2})$. In this work, two particles are randomly selected from the elite set for fitness comparison. The time complexity is O(2n×log(2n)), and the time complexity of learning from the particle with a low fitness value is O(n). For the calculation of the penalty function, the time complexity is $O(n^{2})$, which is dependent on the population size. Each particle is then updated based on an opposition-based learning strategy with a time complexity of O(n). In summary, the time complexity of NMOPSO is $O(m\times n^{2})$. It can be seen that the time complexity is mainly affected by non-dominance sorting. For most MOPSO that need to maintain an external archive, the time complexity is $O(n+n^{2})$, and NMOPSO reduces the time complexity of its archive maintenance by learning from particles with higher fitness values. Therefore, the time complexity of NMOPSO is acceptable.

## 4. Experiment

### 4.1. Experimental Configurations

In this work, NMOPSO’s performance is evaluated by using four pairs of real sensor ontologies, i.e., SN, SSN, OSSN, and SOSA, along with a benchmark test dataset supplied by OAEI. Table 1 and Table 2 offer a concise overview of the sensor ontology and the benchmark test dataset, respectively. The datasets contain reference matching results, source ontology, and target ontology to appraise the performance of the ontology matching system.

**Figure 4:**
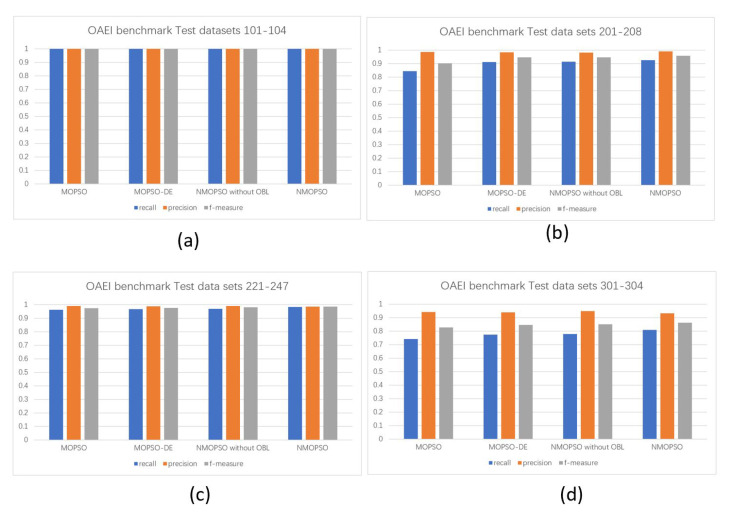
Comparison of recall, precision, and f-measure of different MOPSO-based matching systems on benchmarks.

**Figure 5:**
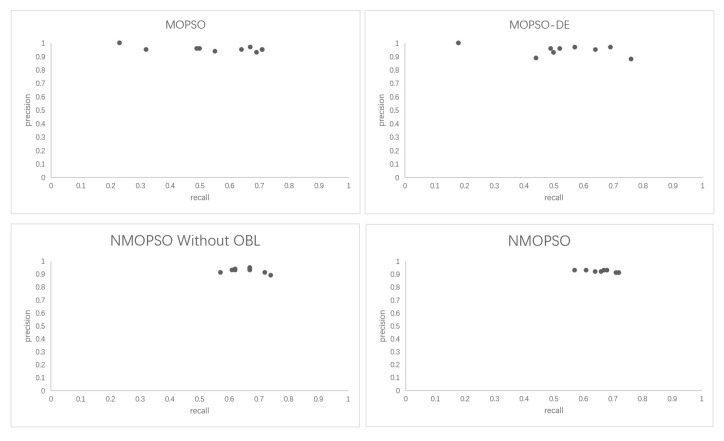
Particle distribution of each matching system on the 301 test dataset.

This experiment firstly verifies the effectiveness of the proposed method. As shown in Table 3, the numbers in brackets are recall, precision, and f-measure. This paper compares the niche-based NMOPSO matching system with the MOPSO-based matching system, the MOPSO-DE matching system based on the diversity-enhancing strategy, and the NMOPSO matching system without OBL. In order to more intuitively verify the effectiveness of the proposed method, Figure 4 shows the comparison between the proposed method and the results of different MOPSO-based matching systems in recall, precision, and f-measure on benchmarks. Table 4 and Table 5 compare the mean and standard deviation of NMOPSO with the MOPSO-based matching technique from recall and precision, respectively. Table 6 and Table 7 show the results of the *t*-test analysis based on Table 4 and Table 5. Table 8 shows the comparison in terms of f-measure among NMOPSO with OAEI participants, i.e., enda [31], AgrMaker [32], AROMA [33], CODI [34], Eff2Match [35], GeRMeSMB [36], MapPSO [37], SOBOM [38], and TaxoMap [39]. The experimental results in this paper are the mean value of the results after 30 independent runs. The metrics of the ontology matching experiment results consists of recall, precision, and f-measure. Recall and precision measure the integrity and accuracy of alignment, respectively. Furthermore, f-measure trades off recall and precision to evaluate the final ontology alignment. Figure 5 shows the distribution of particles in each matching system on the 301 test set. Table 9 and Table 10 show the comparison of the recall and precision of the NMOPSO and classical matching systems on the sensor ontology dataset, respectively.

The configurations of MOPSO and NMOPSO are as follows:

- Population size: *n* = 100;
- Elite particle set scale: *L* = 10;
- Maximum number of iterations: *N* = 200;
- Learning factor: $c_{1}=2$, $c_{2}=2$;
- Upper bound of problem: upp = 1.0;
- Lower bound of problem: low = 0.0;
- Punish quotient: punishQuo = 0.7;
- String-based Similarity Measure: N-Gram;
- Linguistic-based Similarity Measure: Wu and Palmer method;
- Structure-based Similarity Measure: Out–In degree.

The population size of all algorithms in the experiment is set to 100. In addition, the scale of the elite particle set *L* in MOPSO-DE and NMOPSO will affect the convergence and diversity of the population. Usually, a smaller *L* will cause the algorithm to fall into local optima, while a larger one will reduce the algorithm’s convergence. Therefore, this work provides a compromise setting of 10 for *L*. The termination condition for all comparison algorithms is a maximum number of 200 iterations, and each testing case is run separately 30 times. The parameter configurations in this work are derived from the corresponding literature.

### 4.2. Experimental Results

As can be seen from Table 3 and Figure 4, for the testing cases 101–104, all the ontology matching techniques find complete matching pairs and obtain high-quality matching results due to fact that the ontologies of 101–104 have the same lexical, linguistic, and structural features, and the conceptual information of the ontologies is completely retained. For the testing cases 201–247, it is very difficult to find all matching pairs due to the heterogeneity of the ontology, and the NMOPSO-based matching technique can obtain better matching results than other MOPSO-based matching techniques. The ontologies in testing cases 301–304 have more complex heterogeneity than 201–247, but the NMOPSO-based matching technique can achieve better matching results when solving such matching problems in the real world. In particular, to better trade off the two matching objectives, i.e., recall and precision, the NMOPSO-based matching system can find better solutions with better convergence and distribution than the traditional MOPSO-based matching system due to the diversity-enhancing strategy. In addition, since there are multiple global optimal solutions to the ontology meta-matching problem, the traditional MOPSO-based matching system only considers how to balance multiple objectives, while the NMOPSO-based matching system treats the ontology meta-matching problem as a multi-modal problem and seeks multiple global optimal solutions by introducing niching strategies to provide more decision schemes for decision makers and obtain better ontology matching quality. Moreover, to improve the self-learning ability of particles in particle swarm and find solutions with better fitness, NMOPSO introduces the opposition-based learning strategy to further improve the quality of alignments.

Table 4 and Table 5 demonstrate that NMOPSO exhibits high recall and precision on the benchmark dataset and has a low standard deviation. This suggests that NMOPSO has good matching quality and stability. In this work, the *t*-test is used to compare the performance differences between different matching systems. The steps are as follows: first, the mean and standard deviation of 30 independent runs of different matching systems on each testing case are calculated. Second, the t-value is then obtained by dividing the difference between the means of the two samples by the ratio of the standard deviations. The smaller the standard deviation is, the larger the t-value is, indicating the more significant the difference between the two samples is. Finally, the t-value is compared with the rejection domain to determine whether the two samples are significantly different. This paper uses the bilateral test, and the total sample size is 30, with a significance level of 0.05; thus, the rejection region for the *t*-test is |t| ≥ 2.045. When the t-value is in the rejection region, the difference between the two systems is significant, and the null hypothesis is rejected. Otherwise, the null hypothesis is not rejected. It can be seen from Table 6 and Table 7 that the absolute values of all the t-values are greater than 2.045, which indicates that NMOPSO has a significant difference in recall and precision performance compared to other matching systems and can obtain high-quality matching results. Figure 5 shows the distribution of particles between the NMOPSO-based matching system and the MOPSO-based matching system on the 301 test dataset. It can be seen that the population of MOPSO is randomly distributed in the search space, while the population generated by NMOPSO based on the opposition-based learning strategy is able to approach the optimal solution set more closely than MOPSO. Table 8 compares NMOPSO and OAEI participants in terms of f-measure. NMOPSO achieves the highest f-measure in the 1XX and 3XX test cases and has an average f-measure higher than most matching systems, equal to SOMOB. Since SOMOB uses more than three similarity measures, the computational complexity of the system increases and conflict between different measures may occur, which leads to a more challenging matching process. Different from SOMOB, NMOPSO selects and combines three similarity measures, which can achieve satisfactory matching results when solving different heterogeneous situations.

Table 9 and Table 10 demonstrate the comparison between the proposed method based on the sensor ontology dataset and other classic systems in terms of recall and precision, respectively. These systems include Levenshtein-distance-based [40], Jaro–Winkler-distance-based [41], WordNet-similarity-based [42], and Similarity Flooding (SF)-based [43] matching systems. The results demonstrate that the proposed method outperforms or performs as equally well as other sensor ontology matching systems in terms of precision. However, on the SSN-OSSN dataset, the recall is lower than other matching systems, mainly because the matching results of sensor ontologies may involve one-to-many situations, while our method can only identify one-to-one relationships. Additionally, the WordNet dictionary lacks some specialized words related to the sensor domain, which reduces the precision of the matching results. In summary, the proposed NMOPSO-based matching technique in this work can effectively address the sensor ontology meta-matching problem.

## 5. Conclusions

To enable semantic information sharing between sensors, this paper proposed an NMOPSO-based matching technique to effectively address the sensor ontology meta-matching problem. The proposed NMOPSO considers this problem as a multi-modal problem and incorporates a niching strategy to find more optimal solutions in the solution space to meet the diverse needs of decision makers. To improve the diversity of the population, a diversity-enhancing strategy is utilized, and an opposition-based learning strategy is adopted to refine the solutions and improve matching quality. The experimental results demonstrate that the proposed NMOPSO outperforms traditional MOPSO-based matching techniques, as well as participants of OAEI, in terms of effectiveness in solving the sensor meta-ontology matching problem.

Although the proposed matching method can effectively solve the problem of heterogeneity between sensor ontologies when dealing with small-scale sensor ontology matching tasks, the niching strategy can effectively maintain diversity, but it may not be enough to fully explore the search space, particularly if it is large and complex. To overcome this limitation, alternative strategies, such as random initialization or adaptive mutation rates, will be used in the future to encourage broader exploration. Furthermore, analyzing ontology entities and calculating similarity values is typically a time-consuming task. Therefore, a preprocessing strategy for ontology can be implemented to reduce the computational complexity. In the face of large-scale matching tasks, how to effectively and efficiently solve the sensor ontology matching problem is a scientific problem to be solved. In the future, we will focus on the complex semantic relations between ontology entities to make the proposed method suitable for large-scale matching tasks.

## Figures and Tables

**Table 1:** A brief description of OAEI’s benchmark.

ID	Brief Description
1XX	Two ontologies having the same lexical, linguistic, and structural features.
2XX	Two ontologies sharing different structural, lexical, or linguistic features.
3XX	The real ontologies.

**Table 2:** A brief description of sensor ontologies.

Sensor Ontology Information	Scale
SN (SensorOntology2009 ontology)	152 entities
SSN (Semantic Sensor Network ontology)	55 entities
OSSN (Original Semantic Sensor Network ontology)	107 entities
SOSA (Sensor, Observation, Sample, and Actuator ontology)	42 entities

**Table 3:** Comparison of recall, precision, and f-measure among MOPSO, MOPSO-DE, NMOPSO without OBL, and NMOPSO.

No.	MOPSO	MOPSO-DE	NMOPSO without OBL	NMOPSO
101	(1.00, 1.00, 1.00)	(1.00, 1.00, 1.00)	(1.00, 1.00, 1.00)	(1.00, 1.00, 1.00)
103	(1.00, 1.00, 1.00)	(1.00, 1.00, 1.00)	(1.00, 1.00, 1.00)	(1.00, 1.00, 1.00)
104	(1.00, 1.00, 1.00)	(1.00, 1.00, 1.00)	(1.00, 1.00, 1.00)	(1.00, 1.00, 1.00)
201	(0.92, 1.00, 0.96)	(0.92, 1.00, 0.96)	(0.92, 1.00, 0.96)	(0.92, 1.00, 0.96)
203	(1.00, 1.00, 1.00)	(1.00, 1.00, 1.00)	(1.00, 1.00, 1.00)	(1.00, 1.00, 1.00)
204	(0.98, 1.00, 0.99)	(0.98, 1.00, 0.99)	(0.98, 1.00, 0.99)	(0.98, 1.00, 0.99)
205	(0.92, 0.95, 0.94)	(0.92, 0.95, 0.94)	(0.92, 0.95, 0.94)	(0.92, 0.95, 0.94)
206	(0.71, 0.98, 0.82)	(0.86, 0.98, 0.92)	(0.89, 0.95, 0.92)	(0.91, 1.00, 0.95)
207	(0.58, 0.98, 0.73)	(0.87, 0.98, 0.92)	(0.86, 0.98, 0.92)	(0.92, 1.00, 0.96)
208	(0.80, 0.99, 0.88)	(0.83, 0.98, 0.90)	(0.83, 0.98, 0.90)	(0.83, 0.98, 0.90)
221	(1.00, 1.00, 1.00)	(1.00, 1.00, 1.00)	(1.00, 1.00, 1.00)	(1.00, 1.00, 1.00)
222	(1.00, 1.00, 1.00)	(1.00, 1.00, 1.00)	(1.00, 1.00, 1.00)	(1.00, 1.00, 1.00)
223	(0.96, 0.98, 0.96)	(0.94, 0.99, 0.96)	(0.95, 0.99, 0.97)	(0.97, 0.99, 0.98)
224	(1.00, 1.00, 1.00)	(1.00, 1.00, 1.00)	(1.00, 1.00, 1.00)	(1.00, 1.00, 1.00)
225	(1.00, 1.00, 1.00)	(1.00, 1.00, 1.00)	(1.00, 1.00, 1.00)	(1.00, 1.00, 1.00)
228	(1.00, 1.00, 1.00)	(1.00, 1.00, 1.00)	(1.00, 1.00, 1.00)	(1.00, 1.00, 1.00)
230	(0.91, 0.98, 0.95)	(0.97, 0.94, 0.95)	(0.91, 0.98, 0.95)	(0.97, 0.94, 0.96)
231	(1.00, 1.00, 1.00)	(1.00, 1.00, 1.00)	(1.00, 1.00, 1.00)	(1.00, 1.00, 1.00)
232	(1.00, 1.00, 1.00)	(1.00, 1.00, 1.00)	(1.00, 1.00, 1.00)	(1.00, 1.00, 1.00)
233	(1.00, 1.00, 1.00)	(1.00, 1.00, 1.00)	(1.00, 1.00, 1.00)	(1.00, 1.00, 1.00)
236	(1.00, 1.00, 1.00)	(1.00, 1.00, 1.00)	(1.00, 1.00, 1.00)	(1.00, 1.00, 1.00)
237	(1.00, 1.00, 1.00)	(1.00, 1.00, 1.00)	(1.00, 1.00, 1.00)	(1.00, 1.00, 1.00)
238	(0.95, 0.98, 0.96)	(0.92, 0.98, 0.96)	(0.96, 0.98, 0.97)	(0.95, 0.98, 0.97)
240	(0.74, 0.96, 0.82)	(0.81, 0.96, 0.88)	(0.84, 0.96, 0.90)	(0.87, 0.96, 0.92)
241	(1.00, 1.00, 1.00)	(1.00, 1.00, 1.00)	(1.00, 1.00, 1.00)	(1.00, 1.00, 1.00)
246	(1.00, 0.96, 0.98)	(1.00, 0.96, 0.98)	(1.00, 0.96, 0.98)	(1.00, 0.96, 0.98)
247	(0.81, 0.96, 0.88)	(0.81, 0.96, 0.88)	(0.84, 0.96, 0.90)	(0.96, 0.94, 0.95)
301	(0.70, 0.91, 0.79)	(0.70, 0.93, 0.80)	(0.74, 0.91, 0.82)	(0.81, 0.92, 0.86)
302	(0.64, 0.94, 0.76)	(0.65, 0.91, 0.76)	(0.64, 0.96, 0.77)	(0.68, 0.89, 0.77)
303	(0.75, 0.94, 0.83)	(0.79, 0.95, 0.86)	(0.79, 0.95, 0.86)	(0.79, 0.95, 0.86)
304	(0.88, 0.98, 0.93)	(0.96, 0.97, 0.96)	(0.94, 0.97, 0.96)	(0.96, 0.97, 0.96)

**Table 4:** Comparison of mean and standard deviation of recall among MOPSO, MOPSO-DE, NMOPSO without OBL, and NMOPSO.

No.	MOPSO	MOPSO-DE	NMOPSO without OBL	NMOPSO
101	1.00 (0.00)	1.00 (0.00)	1.00 (0.00)	1.00 (0.00)
103	1.00 (0.00)	1.00 (0.00)	1.00 (0.00)	1.00 (0.00)
104	1.00 (0.00)	1.00 (0.00)	1.00 (0.00)	1.00 (0.00)
201	0.92 ($1.22\times 10^{-15}$)	0.92 ($1.08\times 10^{-15}$)	0.92 ($1.08\times 10^{-15}$)	0.92 ($1.08\times 10^{-15}$)
203	1.00 (0.00)	1.00 (0.00)	1.00 (0.00)	1.00 (0.00)
204	0.98 ($2.22\times 10^{-15}$)	0.98 ($2.22\times 10^{-15}$)	0.98 ($2.22\times 10^{-15}$)	0.98 ($2.22\times 10^{-15}$)
205	0.92 ($2.81\times 10^{-1}$)	0.92 ($4.09\times 10^{-1}$)	0.92 ($4.04\times 10^{-1}$)	0.92 ($3.81\times 10^{-1}$)
206	0.71 ($1.14\times 10^{-2}$)	0.86 ($1.10\times 10^{-1}$)	0.89 ($2.41\times 10^{-15}$)	0.91 ($1.90\times 10^{-16}$)
207	0.58 ($2.26\times 10^{-15}$)	0.87 ($1.16\times 10^{-1}$)	0.86 ($1.18\times 10^{-1}$)	0.92 ($1.08\times 10^{-15}$)
208	0.80 ($1.34\times 10^{-2}$)	0.83 ($7.49\times 10^{-2}$)	0.83 ($1.39\times 10^{-2}$)	0.83 ($2.19\times 10^{-2}$)
221	1.00 (0.00)	1.00 (0.00)	1.00 (0.00)	1.00 (0.00)
222	1.00 (0.00)	1.00 (0.00)	1.00 (0.00)	1.00 (0.00)
223	0.96 ($9.27\times 10^{-3}$)	0.94 ($1.51\times 10^{-2}$)	0.95 (0.01)	0.97 ($1.01\times 10^{-2}$)
224	1.00 (0.00)	1.00 (0.00)	1.00 (0.00)	1.00 (0.00)
225	1.00 (0.00)	1.00 (0.00)	1.00 (0.00)	1.00 (0.00)
228	1.00 (0.00)	1.00 (0.00)	1.00 (0.00)	1.00 (0.00)
230	0.91 ($1.34\times 10^{-15}$)	0.97 ($4.03\times 10^{-2}$)	0.91 ($1.03\times 10^{-1}$)	0.97 ($3.23\times 10^{-2}$)
231	1.00 (0.00)	1.00 (0.00)	1.00 (0.00)	1.00 (0.00)
232	1.00 (0.00)	1.00 (0.00)	1.00 (0.00)	1.00 (0.00)
233	1.00 (0.00)	1.00 (0.00)	1.00 (0.00)	1.00 (0.00)
236	1.00 (0.00)	1.00 (0.00)	1.00 (0.00)	1.00 (0.00)
237	1.00 (0.00)	1.00 (0.00)	1.00 (0.00)	1.00 (0.00)
238	0.95 ($5.83\times 10^{-3}$)	0.92 ($5.08\times 10^{-15}$)	0.96 ($1.33\times 10^{-3}$)	0.95 ($1.01\times 10^{-2}$)
240	0.74 ($1.27\times 10^{-1}$)	0.81 ($1.33\times 10^{-1}$)	0.84 ($3.59\times 10^{-2}$)	0.87 ($1.30\times 10^{-2}$)
241	1.00 (0.00)	1.00 (0.00)	1.00 (0.00)	1.00 (0.00)
246	1.00 ($4.33\times 10^{-1}$)	1.00 ($2.79\times 10^{-1}$)	1.00 ($3.81\times 10^{-1}$)	1.00 ($4.54\times 10^{-1}$)
247	0.81 ($3.02\times 10^{-1}$)	0.81 ($1.16\times 10^{-1}$)	0.84 ($1.66\times 10^{-1}$)	0.96 ($1.92\times 10^{-1}$)
301	0.70 ($9.84\times 10^{-2}$)	0.70 ($1.13\times 10^{-1}$)	0.74 ($9.36\times 10^{-2}$)	0.81 ($1.91\times 10^{-2}$)
302	0.64 ($8.71\times 10^{-16}$)	0.65 ($1.59\times 10^{-2}$)	0.64 ($9.04\times 10^{-3}$)	0.68 ($1.95\times 10^{-2}$)
303	0.75 ($1.03\times 10^{-15}$)	0.79 ($3.45\times 10^{-2}$)	0.79 ($3.60\times 10^{-2}$)	0.79 ($2.47\times 10^{-2}$)
304	0.88 ($8.29\times 10^{-4}$)	0.96 ($1.37\times 10^{-1}$)	0.94 ($3.62\times 10^{-2}$)	0.96 ($3.59\times 10^{-2}$)

**Table 5:** Comparison of mean and standard deviation of precision among MOPSO, MOPSO-DE, NMOPSO without OBL, and NMOPSO.

No.	MOPSO	MOPSO-DE	NMOPSO without OBL	NMOPSO
101	1.00 (0.00)	1.00 (0.00)	1.00 (0.00)	1.00 (0.00)
103	1.00 (0.00)	1.00 (0.00)	1.00 (0.00)	1.00 (0.00)
104	1.00 (0.00)	1.00 (0.00)	1.00 (0.00)	1.00 (0.00)
201	1.00 (0.00)	1.00 (0.00)	1.00 (0.00)	1.00 (0.00)
203	1.00 (0.00)	1.00 (0.00)	1.00 (0.00)	1.00 (0.00)
204	1.00 (0.00)	1.00 (0.00)	1.00 (0.00)	1.00 (0.00)
205	0.95 ($1.45\times 10^{-2}$)	0.95 ($2.11\times 10^{-2}$)	0.95 ($2.08\times 10^{-2}$)	0.95 ($1.96\times 10^{-2}$)
206	0.98 ($1.91\times 10^{-4}$)	0.98 ($5.86\times 10^{-3}$)	0.95 ($1.75\times 10^{-2}$)	1.00 (0.00)
207	0.98 ($1.63\times 10^{-15}$)	0.98 ($5.21\times 10^{-3}$)	0.98 ($4.91\times 10^{-3}$)	1.00 (0.00)
208	0.99 ($2.81\times 10^{-3}$)	0.98 ($5.76\times 10^{-3}$)	0.98 ($2.13\times 10^{-4}$)	0.98 ($1.96\times 10^{-3}$)
221	1.00 (0.00)	1.00 (0.00)	1.00 (0.00)	1.00 (0.00)
222	1.00 (0.00)	1.00 (0.00)	1.00 (0.00)	1.00 (0.00)
223	0.98 ($9.46\times 10^{-3}$)	0.99 ($1.02\times 10^{-2}$)	0.99 ($5.19\times 10^{-3}$)	0.99 ($5.20\times 10^{-3}$)
224	1.00 (0.00)	1.00 (0.00)	1.00 (0.00)	1.00 (0.00)
225	1.00 (0.00)	1.00 (0.00)	1.00 (0.00)	1.00 (0.00)
228	1.00 (0.00)	1.00 (0.00)	1.00 (0.00)	1.00 (0.00)
230	0.98 ($1.45\times 10^{-15}$)	0.94 ($2.38\times 10^{-2}$)	0.98 ($5.61\times 10^{-3}$)	0.94 ($1.70\times 10^{-2}$)
231	1.00 (0.00)	1.00 (0.00)	1.00 (0.00)	1.00 (0.00)
232	1.00 (0.00)	1.00 (0.00)	1.00 (0.00)	1.00 (0.00)
233	1.00 (0.00)	1.00 (0.00)	1.00 (0.00)	1.00 (0.00)
236	1.00 (0.00)	1.00 (0.00)	1.00 (0.00)	1.00 (0.00)
237	1.00 (0.00)	1.00 (0.00)	1.00 (0.00)	1.00 (0.00)
238	0.98 ($5.63\times 10^{-3}$)	0.98 ($1.64\times 10^{-15}$)	0.98 ($8.74\times 10^{-3}$)	0.98 ($5.23\times 10^{-3}$)
240	0.96 ($1.66\times 10^{-2}$)	0.96 ($4.29\times 10^{-2}$)	0.96 ($1.70\times 10^{-2}$)	0.96 ($1.57\times 10^{-2}$)
241	1.00 (0.00)	1.00 (0.00)	1.00 (0.00)	1.00 (0.00)
246	0.96 ($1.55\times 10^{-2}$)	0.96 ($1.00\times 10^{-2}$)	0.96 ($1.36\times 10^{-2}$)	0.96 ($1.62\times 10^{-2}$)
247	0.96 ($1.61\times 10^{-2}$)	0.96 ($1.69\times 10^{-2}$)	0.96 ($1.67\times 10^{-2}$)	0.94 ($2.83\times 10^{-2}$)
301	0.91 ($1.05\times 10^{-2}$)	0.93 ($2.52\times 10^{-2}$)	0.91 ($1.77\times 10^{-2}$)	0.92 ($4.23\times 10^{-3}$)
302	0.94 ($1.22\times 10^{-15}$)	0.91 ($3.02\times 10^{-2}$)	0.96 ($4.51\times 10^{-2}$)	0.89 ($2.71\times 10^{-2}$)
303	0.94 ($1.12\times 10^{-15}$)	0.95 ($2.36\times 10^{-2}$)	0.95 ($2.34\times 10^{-2}$)	0.95 ($1.60\times 10^{-2}$)
304	0.98 ($1.34\times 10^{-5}$)	0.97 ($7.33\times 10^{-3}$)	0.97 ($1.08\times 10^{-2}$)	0.97 ($7.85\times 10^{-3}$)

**Table 6:** T-test statistical analysis for recall.

No.	NMOPSO versus MOPSO	NMOPSO versus MOPSO-DE	NMOPSO versus NMOPSO without OBL
101	0.00	0.00	0.00
103	0.00	0.00	0.00
104	0.00	0.00	0.00
201	0.00	0.00	0.00
203	0.00	0.00	0.00
204	0.00	0.00	0.00
205	0.00	0.00	0.00
206	96.09	2.48	4.53
207	7.43	2.36	2.78
208	6.40	0.00	0.00
221	0.00	0.00	0.00
222	0.00	0.00	0.00
223	3.99	9.04	7.70
224	0.00	0.00	0.00
225	0.00	0.00	0.00
228	0.00	0.00	0.00
230	10.17	0.00	3.04
231	0.00	0.00	0.00
232	0.00	0.00	0.00
233	0.00	0.00	0.00
236	0.00	0.00	0.00
237	0.00	0.00	0.00
238	0.00	16.26	−5.37
240	5.57	2.45	4.30
241	0.00	0.00	0.00
246	0.00	18.45	0.00
247	2.29	3.66	2.58
301	6.01	5.25	4.01
302	11.23	6.53	10.19
303	8.87	0.00	0.00
304	12.20	0.00	2.14

**Table 7:** T-test statistical analysis for precision.

No.	NMOPSO versus MOPSO	NMOPSO versus MOPSO-DE	NMOPSO versus NMOPSO without OBL
101	0.00	0.00	0.00
103	0.00	0.00	0.00
104	0.00	0.00	0.00
201	0.00	0.00	0.00
203	0.00	0.00	0.00
204	0.00	0.00	0.00
205	0.00	0.00	0.00
206	573.53	18.69	15.64
207	6.72	21.02	22.31
208	−15.98	0.00	0.00
221	0.00	0.00	0.00
222	0.00	0.00	0.00
223	5.07	0.00	0.00
224	0.00	0.00	0.00
225	0.00	0.00	0.00
228	0.00	0.00	0.00
230	−12.88	0.00	−12.23
231	0.00	0.00	0.00
232	0.00	0.00	0.00
233	0.00	0.00	0.00
236	0.00	0.00	0.00
237	0.00	0.00	0.00
238	0.00	0.00	0.00
240	0.00	0.00	0.00
241	0.00	0.00	0.00
246	0.00	0.00	0.00
247	−1.36	−1.32	−1.33
301	4.83	−1.14	3.00
302	−10.10	−1.62	−1.50
303	3.42	0.00	0.00
304	−1.97	0.00	0.00

**Table 8:** Comparison of NMOPSO with OAEI’s participants in terms of f-measure.

No.	Edna	AgrMaker	AROMA	CODI	Ef2Match	GeRMeSMB	MapPSO	SOBOM	TaxoMap	NMOPSO
101	1.00	0.99	0.98	1.00	1.00	1.00	1.00	1.00	0.51	1.00
103	1.00	0.99	0.99	1.00	1.00	1.00	1.00	1.00	0.51	1.00
104	1.00	0.99	0.99	0.99	1.00	1.00	1.00	1.00	0.51	1.00
201	0.04	0.92	0.95	0.13	0.77	0.94	0.42	0.95	0.51	0.96
203	1.00	0.98	0.80	0.86	1.00	0.98	1.00	1.00	0.49	1.00
204	0.93	0.97	0.97	0.74	0.99	0.98	0.98	0.99	0.51	0.99
205	0.34	0.92	0.95	0.28	0.84	0.99	0.73	0.96	0.51	0.94
206	0.54	0.93	0.95	0.39	0.87	0.92	0.85	0.96	0.51	0.95
207	0.54	0.93	0.95	0.42	0.87	0.96	0.81	0.96	0.51	0.96
208	0.93	0.96	0.58	0.61	0.95	0.95	0.79	1.00	0.44	0.90
221	1.00	0.97	0.99	0.98	1.00	1.00	1.00	1.00	0.51	1.00
222	0.98	0.98	0.99	1.00	1.00	0.99	1.00	1.00	0.46	1.00
223	1.00	0.95	0.93	1.00	1.00	0.96	0.98	0.99	0.45	0.98
224	1.00	0.99	0.97	1.00	1.00	1.00	1.00	1.00	0.51	1.00
225	1.00	0.99	0.99	0.99	1.00	1.00	1.00	1.00	0.51	1.00
228	1.00	1.00	1.00	1.00	1.00	1.00	1.00	1.00	0.51	1.00
230	0.85	0.90	0.93	0.98	0.97	0.94	0.98	0.97	0.49	0.96
231	1.00	0.99	0.98	1.00	1.00	1.00	1.00	0.97	0.51	1.00
232	1.00	0.97	0.97	0.97	1.00	1.00	1.00	1.00	0.51	1.00
233	1.00	1.00	1.00	0.94	1.00	0.98	1.00	1.00	1.00	1.00
236	1.00	1.00	1.00	1.00	1.00	1.00	1.00	1.00	1.00	1.00
237	0.98	0.98	0.97	0.99	1.00	1.00	0.99	1.00	0.46	1.00
238	1.00	0.94	0.92	0.99	1.00	0.96	0.97	0.98	0.45	0.97
240	0.55	0.91	0.83	0.95	0.98	0.85	0.92	0.98	0.88	0.92
241	1.00	1.00	0.98	0.94	1.00	0.98	1.00	1.00	1.00	1.00
246	0.50	0.98	0.97	0.98	0.98	0.98	0.98	0.95	0.94	0.98
247	0.55	0.88	0.80	0.98	0.98	0.91	0.89	0.98	0.88	0.95
301	0.59	0.59	0.73	0.38	0.71	0.71	0.64	0.84	0.43	0.86
302	0.43	0.32	0.35	0.59	0.71	0.41	0.04	0.74	0.40	0.77
303	0.00	0.78	0.59	0.65	0.83	0.00	0.00	0.50	0.36	0.86
304	0.83	0.86	0.84	0.74	0.95	0.77	0.72	0.91	0.52	0.96
Ave	0.79	0.92	0.90	0.82	0.95	0.91	0.86	0.96	0.57	0.96

**Table 9:** Comparison of recall of NMOPSO with classical matching systems on the sensor ontology dataset.

Sensor Ontology Alignment Task	Levenshtein-Distance -Based Matcher	Jaro–Winkler-Distance -Based Matcher	WordNet-Similarity -Based Matcher	SF-Based Matcher	NMOPSO
SSN-SN	1.00	1.00	1.00	0.56	1.00
SOSA-SN	1.00	1.00	1.00	1.00	1.00
SOSA-OSSN	1.00	1.00	1.00	0.50	1.00
SSN-OSSN	1.00	1.00	0.97	0.35	0.93

**Table 10:** Comparison of precision of NMOPSO with classical matching systems on the sensor ontology dataset.

Sensor Ontology Alignment Task	Levenshtein-Distance -Based Matcher	Jaro–Winkler-Distance -Based Matcher	WordNet-Similarity -Based Matcher	SF-Based Matcher	NMOPSO
SSN-SN	1.00	1.00	1.00	0.56	1.00
SOSA-SN	1.00	1.00	1.00	1.00	1.00
SOSA-OSSN	1.00	1.00	1.00	0.50	1.00
SSN-OSSN	1.00	1.00	0.97	0.35	1.00

## Data Availability

The data in this work can be obtained from the corresponding author upon request.
